# *iMSRC*: converting a standard automated microscope into an intelligent screening platform

**DOI:** 10.1038/srep10502

**Published:** 2015-05-27

**Authors:** Angel Carro, Manuel Perez-Martinez, Joaquim Soriano, David G. Pisano, Diego Megias

**Affiliations:** 1 Bioinformatics unit, Structural Biology and Biocomputing Programme, Spanish National Cancer Research Centre (CNIO), Madrid, Spain; 2 Confocal Microscopy Unit, Biotechnology programme, CNIO, Madrid, Spain

## Abstract

Microscopy in the context of biomedical research is demanding new tools to automatically detect and capture objects of interest. The few extant packages addressing this need, however, have enjoyed limited uptake due to complexity of use and installation. To overcome these drawbacks, we developed *iMSRC*, which combines ease of use and installation with high flexibility and enables applications such as rare event detection and high-resolution tissue sample screening, saving time and resources.

High automation capabilities applied to optical microscopy paved the way for converting microscopes into intelligent screening platforms, i.e. instruments able to automatically detect objects of interest based on an image analysis routine and capture them at high resolution. The use of this new strategy saves on acquisition time, optimizes resources (Fig. 1 and Fig. 2b and c and Supplementary Fig. S5) and enables advanced applications, such as rare event detection (e.g. circulating tumour cells (CTCs); Fig. 1 and Fig. 2c).

While there are a small number of open-source codes which might permit microscope automation (see, e.g. Icy^1^, Endrov^2^ or Micropilot^3^), these usually require advanced programming skills and are often hobbled by hardware compatibility issues (e.g. driver availability on the Micro-manager^4^ platform), making their implementation difficult for core facilities and, more importantly, being complex to use and adapt.

On the other hand, microscope manufacturers’ acquisition software is much easier to use and boasts optimized hardware compatibility. However, due to its very limited flexibility, its use in intelligent screening approaches is often unfeasible.

To overcome these limitations, we developed ***iMSRC*** (intelligent Matrix Screener Remote Control), a new open source software-based solution capable of implementing distinct intelligent screening applications through exploiting the simplicity of the microscope’s native software (Supplementary Fig. S1).

***iMSRC*** gathers all relevant information from acquisition files generated by proprietary software. Then, upon analyzing preliminary images (“First scan”) through conventional image analysis platforms (e.g. ImageJ^5^ or MATLAB) it creates new acquisition files containing ad hoc generated settings, managing the launch of a new capture sequence focused on objects of interest, meeting the specific requirements of any experimental design, and allowing for complex experimental set-up not otherwise possible (Fig. 1 and Supplementary Fig. S1). Symbiosis with proprietary software guarantees hardware compatibility (there is no need for installing new pieces of hardware or drivers, or programming new drivers), ease of use (there is no need to learn to use a new capturing environment) and avoids the need for complex hardware and software installation.

***iMSRC***-driven microscopy is user-friendly, fully compatible with both confocal and wide-field microscopes and accessible to any user, independently of their programming or microscopy skills.

***iMSRC*** has already proven its functionality and versatility in several research applications, including high-resolution Tissue Microarray confocal capture^6^ (Fig. 2b), live, time-lapse, single stem cell detection in customized PDMS microchips^7^,^8^, hippocampus dentate gyrus-specific cell-type detection in brain sections (Supplementary Fig. S6) and circulating tumour cell (CTCs) detection^9^ (Fig. 2c). The identification and high-magnification mosaic capturing of specific regions within tissue sections, such as in the case of breast metastasis and GFP-expressing regions in different organs have also been successfully accomplished.

***iMSRC*** can be deployed in any conventional Linux PC following a few simple installation steps, publishing the user graphical interface for software control as a web page, which is then fully accessible through the most common web browsers from any computer within the same network (Supplementary Fig. S1b). The addition of new microscopes to the structure is straightforward, rapid and can be performed within the same web page (Supplementary Fig. S2).

Although direct connection with a microscope workstation is also possible, network access offers the following advantages, which are particularly useful to core facilities and laboratories that share equipment: (i) a single ***iMSRC*** installation can be accessed by all instruments, managing intelligent screening in all of them, unifying access and simplifying use; (ii) access to all required utilities, including image analysis script routines and microscope choice dialog, is centralized, making management easier; (iii) to ensure safety, all software administration (including macro upload, see Supplementary Fig. S3, or a new microscope addition, see Supplementary Fig. S2) can be made accessible only to authorized staff using authentication. Besides the novelty of this approach, no additional software installation on the microscope computers is required, maintaining maximum performance.

***iMSRC***’s full compatibility has been thoroughly assessed in different institutions and on a wide variety of systems and local network configurations.

***iMSRC*** has been tested on Leica TCS-SP5 and TCS-SP8 laser scanning confocals and in Leica DMI6000B and DMI8 wide-field microscopes. The only requisite for iMSRC to work is that the proprietary software running a microscope generates a comprehensive metadata file that can be edited and loaded again into such software. For this reason, compatibility with Nikon and Zeiss does not seem plausible to date; compatibility with any other microscopes (e.g. Olympus) is conditioned to the fact that the previous criteria are fulfilled.

How to use ***iMSRC***

Intelligent screenings with ***iMSRC*** are performed in three steps (Figs. 1,2 and Supplementary Fig. S6).

Step 1. First scan: capture of a searching mosaic, composed of juxtaposed images covering the entire sample. This is intended to have minimum resolution, with a large field of view in order to locate the events of interest at the maximum possible speed.

Step 2. Running an image analysis routine designed to locate the coordinates of the objects of interest previously defined in the searching mosaic

Step 3. Second scan: Final acquisition of identified objects using the parameter settings predefined to achieve the desired image properties (i.e. objective, zoom, Z-stack and time lapse). Importantly, the change of objective lenses between scans will be directly controlled by ***iMSRC***, which automatically applies predefined paracentricity correction (Supplementary Fig. S4).

***iMSRC*** supports multiple sample acquisition and allows any format compatible with a particular microscope’s frame adapters, such as microtiter plates and dishes, or up to five slides in parallel (Supplementary Fig. S6).

Parameters for both first and second scans are built up in the proprietary software and must be stored as two independent files in a local or remote shared folder (Supplementary Fig. S2).

***iMSRC*** is equipped by default with a series of macros for tissue or cell detection which covers the vast majority of applications. If these are not sufficient to implement a particular object detection, a user with no programming skills but basic knowledge of image analysis could create his or her own image analysis routine and simply copy and paste one line of code in order to make it fully compatible with ***iMSRC*** (Supplementary Fig. S3).

New image analysis routines can be virtually, designed using any image analysis software, as long as the final result is a list of coordinates of detected objects. Additionally, objects of interest can also be manually selected from the searching mosaic image by the user, adding a further layer of useful flexibility to the whole system (Supplementary Fig. S6).

The ***iMSRC*** graphical interface is accessible through a web page where the user can easily choose among different options, allowing for loading the previously saved capture settings for both first and second scans on extendable menus and fine-tuning of all parameters necessary for objects detection (Fig. 2a and Supplementary Fig. S3).

Once all the settings have been defined, they can be saved as a template and reloaded, which simplifies further experiments.

When ***iMSRC*** is launched, objects smaller than a single field of view will be captured as an individual picture; bigger objects will be covered by high-resolution mosaics. In the case of the latter, the acquisition of non-informative regions (NoIR) in the vicinity of the objects of interest can be prevented by activating the “NoIR” option (Fig. 2a). When this additional feature is activated, a secondary analysis for just those objects identified in Step 2 will run, disabling irrelevant acquisition fields. This ensures the perfect fitting for irregular samples, saving valuable acquisition time and resources — something that is particularly relevant when capturing large, irregularly-shaped objects (Supplementary Fig. S5).

If a microscope is equipped with a communications port (i.e. the Computer Aided Microscope port [CAM] of Leica microscopes), ***iMSRC*** can automatically launch the whole process that directs information flow between the capturing and analysis software by means of a single click (“Play”; Supplementary Fig. S1). Importantly, even when this port is not available, each step can still be sequentially launched by the user. Manual workflow is also recommended when performing complex image analysis, which might require a revision of the identified areas prior to launching the final acquisition step.

## Conclusions

We present ***iMSRC***, a tool based on a new approach that converts conventional automated microscopes into easy-to-use intelligent systems able to automatically localize and capture events of interest, making novel experimental designs affordable.

iMSRC’s has already proved its applicability thorough its implementation in several papers publications and its spreading to different institutions. The combination of features offered by the iMSRC platform make it a valuable tool, which fills a wide gap in the field of bioimaging software usability, as identified in a recent publication^10^.

***IMSRC*** download and more details are available in http://***iMSRC***.bioinfo.cnio.es web page.

## Materials and Methods

In all cases, images were acquired using a confocal TCS-SP5-WLL (AOBS) spectral microscope (Leica Mycrosystems, Wetzlar, Germany). Its acquisition software is LAS AF v2.6 (Leica).

### Automated, multiple, size- and shape-heterogeneous, tissue microarray capturing (Fig. 2b).

This is based on previously published work — check reference 7 for further details.

Several control and clinical tissues can be extracted, fixed, cut into small pieces and placed on the same slide for further staining. The result is called a Tissue Microarray (TMA), which is useful for minimizing sample variation and use of reagents.

Briefly, TMAs consisting of a mixture of healthy and cancerous tissue types and phases were obtained from mouse xenograft models, double immunostained for different pairs of primary antibodies, revealed with Alexa Fluor 488 and Alexa Fluor 555, and nucleus-counterstained with DAPI.

The resulting sample was analyzed under a microscope driven by ***iMSRC*** which was tuned to automatically detect and capture at high resolution every single region of tissue in the TMA.

The acquisition workflow is detailed in supplementary materials and methods.

### Automated image analysis detection of rare circulating tumour cells (Fig. 2c).

Circulating tumour cells (CTCs) spread to the blood from both primary and metastatic cancers, and are believed to play a role in the spread of the disease throughout the body^9^.

A novel microfluidic device (The IsoFlux System, Fluxion Biosciences Inc, South San Francisco,CA) has recently been described and which enables the CTC enrichment of blood samples. These samples are usually further tested using an immunofluorescence assay based on detecting nucleated, cytokeratin (CK)-positive, CD45-negative cells.

Detailed protocols can be found in reference 9 and in Fluxion Biosciences Inc’s CTC Enumeration and CTC Enrichment kits.

A typical analytical design for testing CTC recovery in this device begins with preparing samples with a defined number of CTCs, which is done by isolating a known number of positive CTCs and spiking them into blood from a healthy donor. The frequency of CTCs in these samples is generally less than 1 in 400 (0.25%), which justifies the need for automated cell detection.

Briefly, clinical blood samples were processed for CTC enrichment, subsequently spiked into healthy donor blood and dispensed on to a standard slide for imaging. Cell nuclei were then counterstained with Hoechst 33342 and double immunostained for CK (FITC directly conjugated fluorophore) and CD45 (indirectly labeled with a Cy3-conjugated antibody).

The resulting sample was analyzed under a microscope driven by ***iMSRC*** set to automatically detect and capture CTCs at high resolution.

The acquisition workflow is detailed in supplementary materials and methods.

## Additional Information

**How to cite this article**: Carro, A. *et al*. *iMSRC*: converting a standard automated microscope into an intelligent screening platform. *Sci. Rep.* **5**, 10502; doi: 10.1038/srep10502 (2015).

## Supplementary Material

Supplementary Information

## Figures and Tables

**Figure 1:**
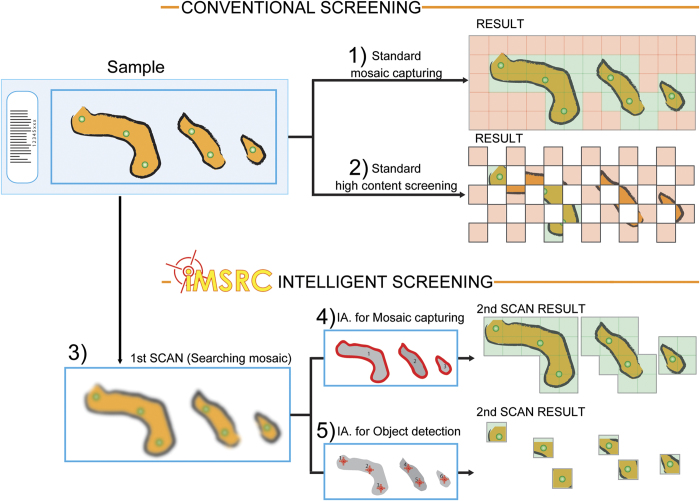
Standard mosaic capturing (1) and high content screening (2) compared to *IMSRC*’s intelligent screening applied to mosaic capturing (4) and object detection(5). ***iMSRC*** uses a three-step approach: capturing a searching mosaic through a first scan (3), extracting objects of interest’s coordinates by using image analysis routines (4 and 5) and ,finally, using these coordinates for a second scan (4 and 5, right column images). ***iMSRC*** increases capturing efficiency by preventing non-informative-area capturing (light red insets on 1 and 2, right column images). Light green insets on right column images show final resolution, meaningful captured areas.

**Figure 2:**
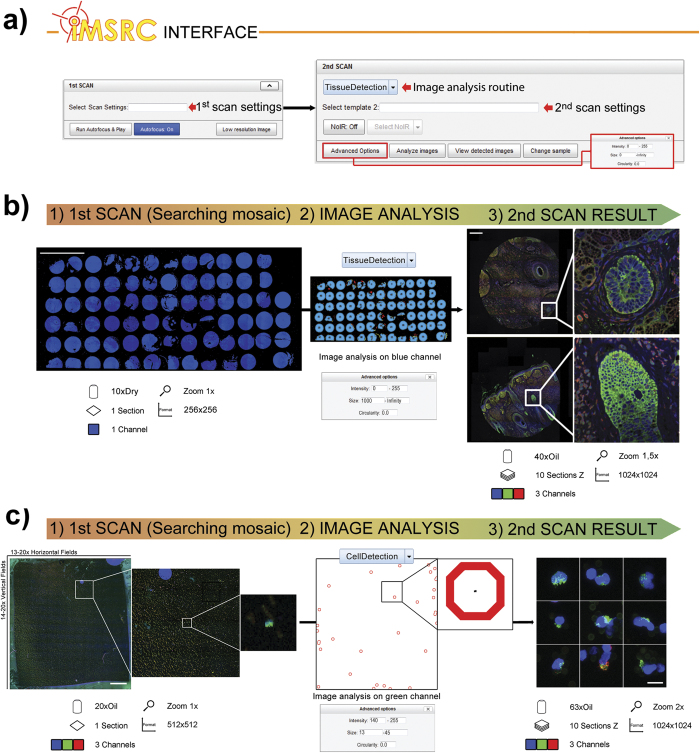
*iMSRC* user’s interface (a), *iMSRC* applied to mosaic capturing (b) and detection of rare events (c). ***iMSRC*** allows the automatic capturing of complex experimental designs by choosing previously saved files on extendable menus, and tuning three simple image analysis parameters (**a**). Searching mosaic (b1 and c1), image-analysis-detected objects (b2 and c2) and final, merged-colour maximum projections (b3 and c3) are shown. Seventy-seven differently sized tissue pieces belonging to a Tissue Microarray were automatically captured overnight in (**b**). Circulating Tumour Cells (1:400) were automatically detected in (**c**). Insets show details. Text and icons show step specifics; see the Material and Methods section for further details. Scale bars (b1): 5 mm; (b3): 250 microns; (c1): 600 microns; and (c3): 12 microns.
