# Supplementary material for: iMSRC: converting a standard automated microscope into an intelligent screening platform
Source: Sci Rep. 2015 May 27;5:10502. doi: 10.1038/srep10502 (PMC4444834; doi:10.1038/srep10502)
Supplement: Supplementary Information [file srep10502-s1.pdf]

Supplementary Information to:

iMSRC: converting a standard automated microscope into an intelligent screening platform

Angel Carro[1¶], Manuel Perez-Martinez[2¶], Joaquim Soriano[2¶], David G. Pisano[1], Diego Megias[2]

Supplementary Figures

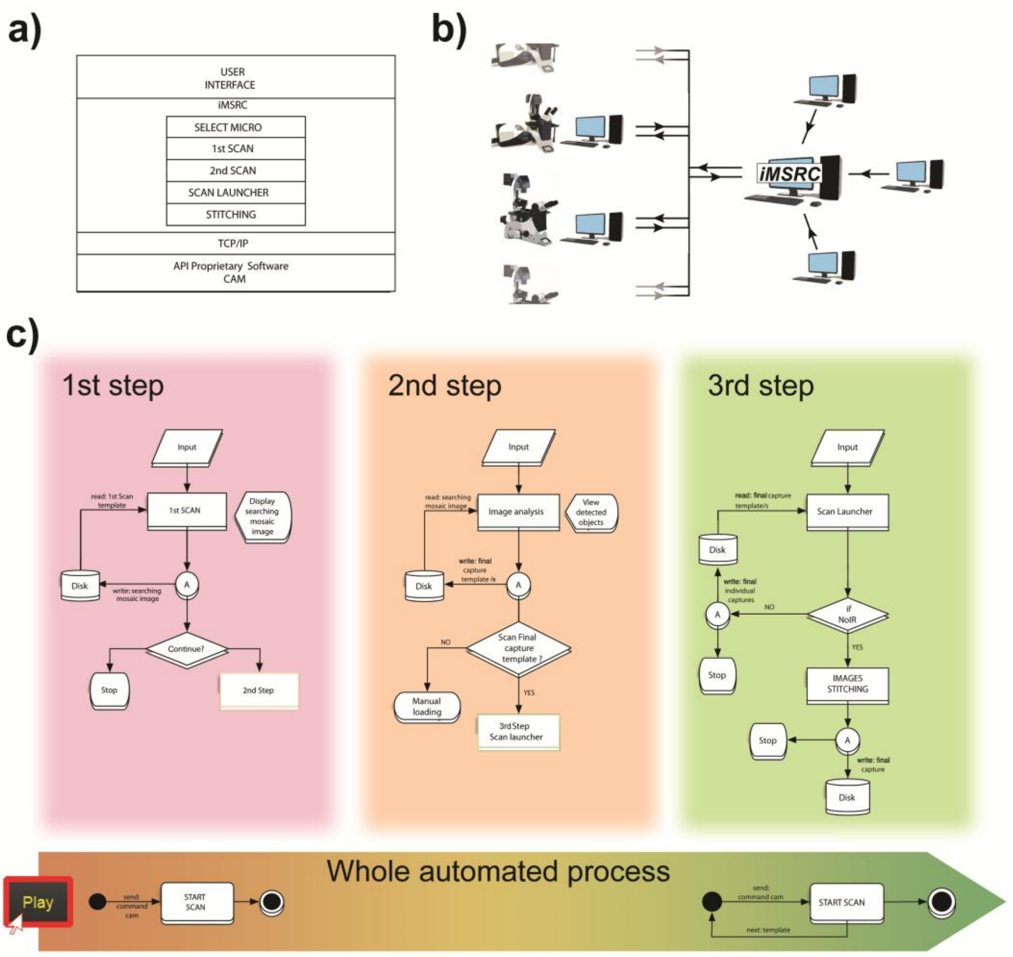

Supplementary Figure S1: *iMSRC*'s software architecture (a), network setup (b) and data flow diagram (c). *iMSRC* user's input and control is performed via a web interface built using HTML and Javascript, image acquisition and storage, files organization and image analysis is done using custom Perl scripting and the communication with the microscope's computer is done through TCP/IP. An optional CAM module allows for direct microscope's communication through proprietary API (a) automating the whole process (c). ***iMSRC*** can be accessed from different computers and manage several microscopes (b).

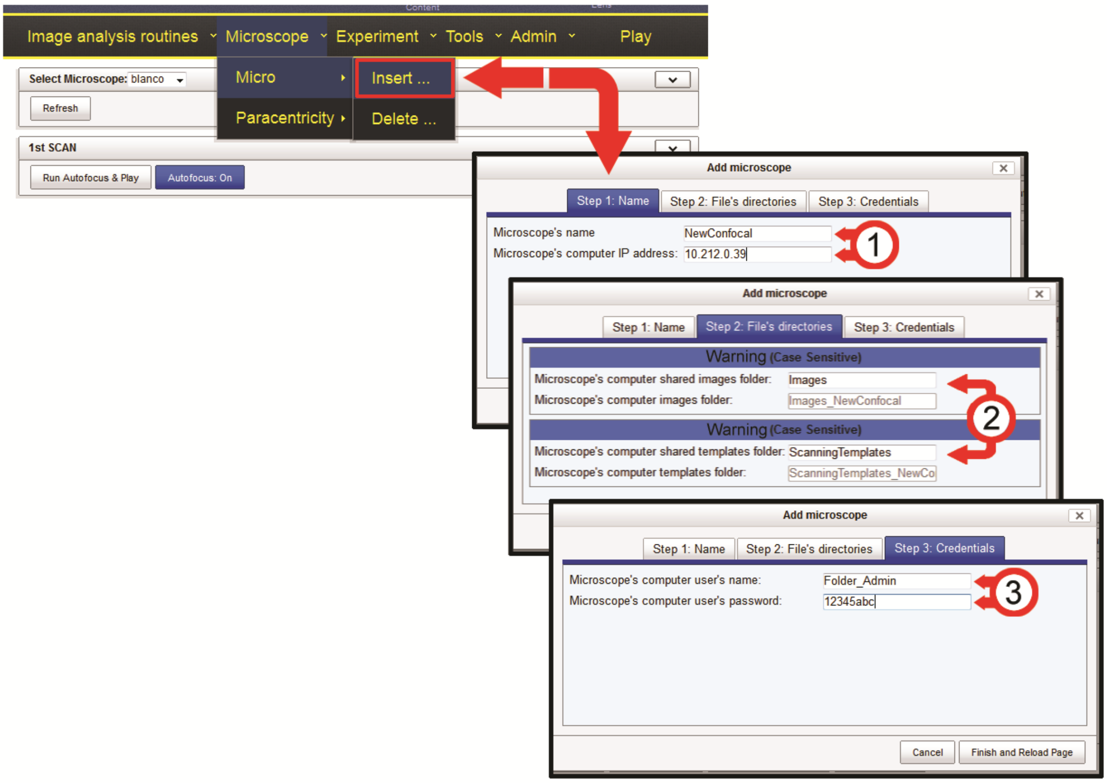

Supplementary Figure S2: *iMSRC* new microscope addition menu. Setting up ***iMSRC*** to work with a new microscope can be accomplished in three easy steps by providing effortless data about the computer managing of the equipment: a name that will identify the microscope, the microscope's computer IP address (1), the name of the folder in which the images and the settings´ templates are stored (both folders should be shared with ***iMSRC***'s computer to grant accessibility) (2) and a microscope's computer user's name and password (3).

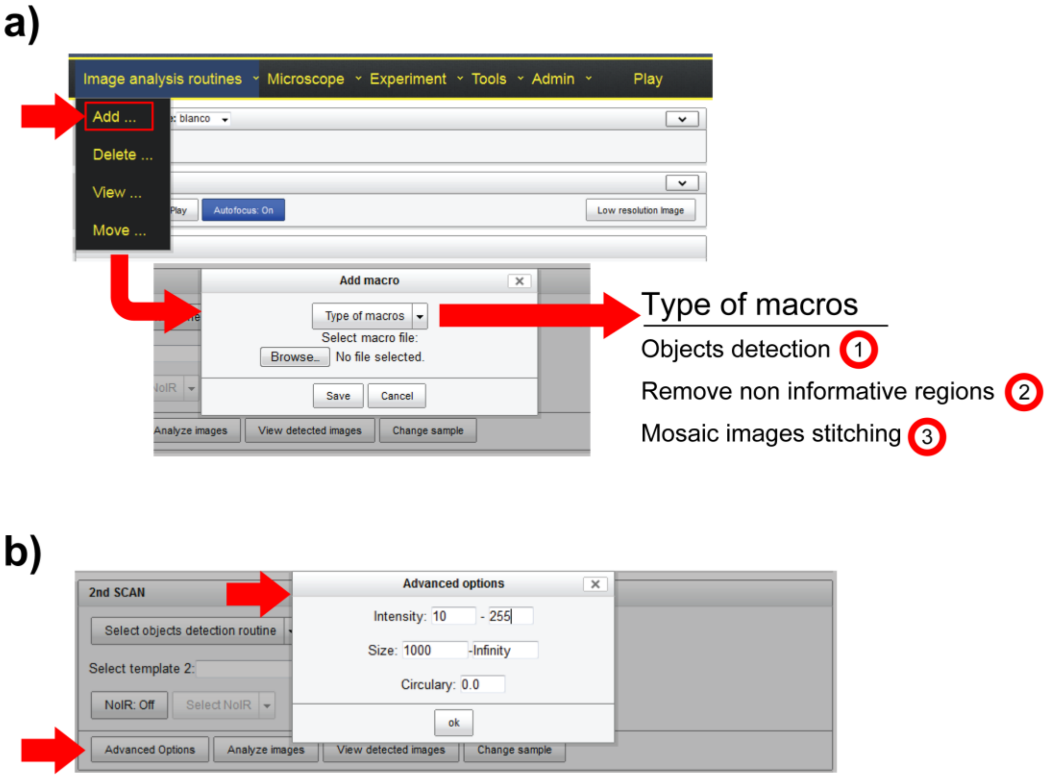

Supplementary Figure S3: *iMSRC*'s Image analysis routines and Image analysis advanced options menus. Previously designed image analysis routines to be used through ***iMSRC***'s extendable menus can be managed through a specific menu that will classify them upon its use (a). A simple image j- designed object detection macro is provided that will suffice for most object detection applications. It is based on three simple parameters (object's signal intensity, size and circularity) that can be interactively modified and checked during ***iMSRC***'s image analysis step (b). For compatibility with ***iMSRC*** new macros only need to include a first line with "*open("#file#");*" instruction.

a)

Parcentricity correction on *iMSRC*

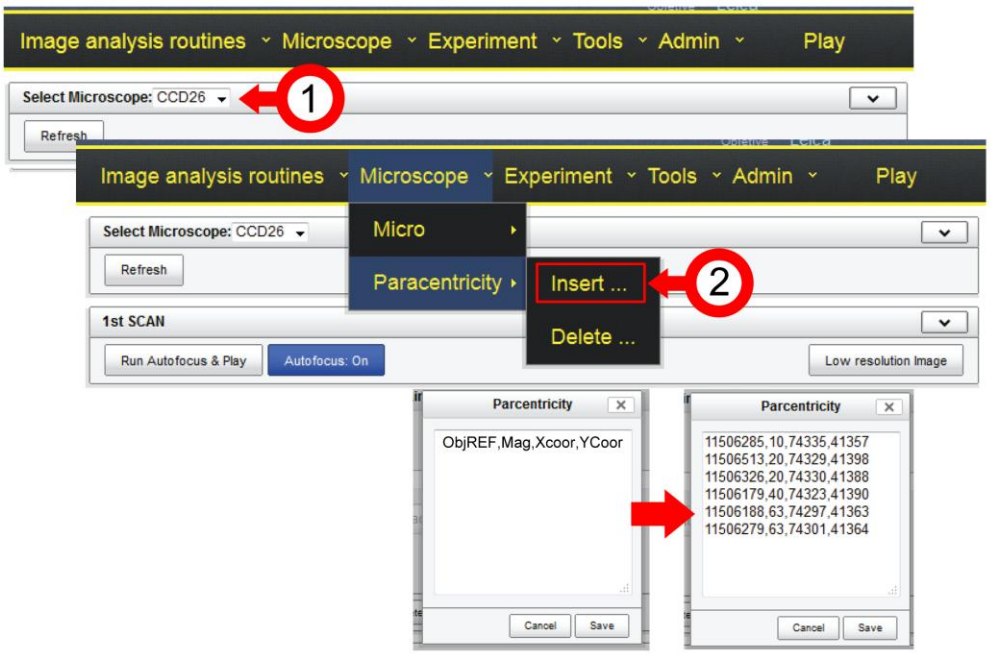

b)

Parcentricity correction

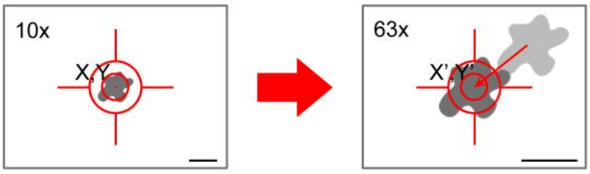

Supplementary Figure S4: *iMSRC* objective's paracentricity correction process. Paracentricity can be easily corrected through ***iMSRC*** Paracentricity menu by providing simple data about the objective's to be used in the first and second scans: objective's reference name (to be read from the capturing software or the objective itself), objective's magnification and x and y object's coordinates (a) (to be read from the capturing software after focusing the same object under each objective) (b). This step can be skipped if the equipment is paracentric.

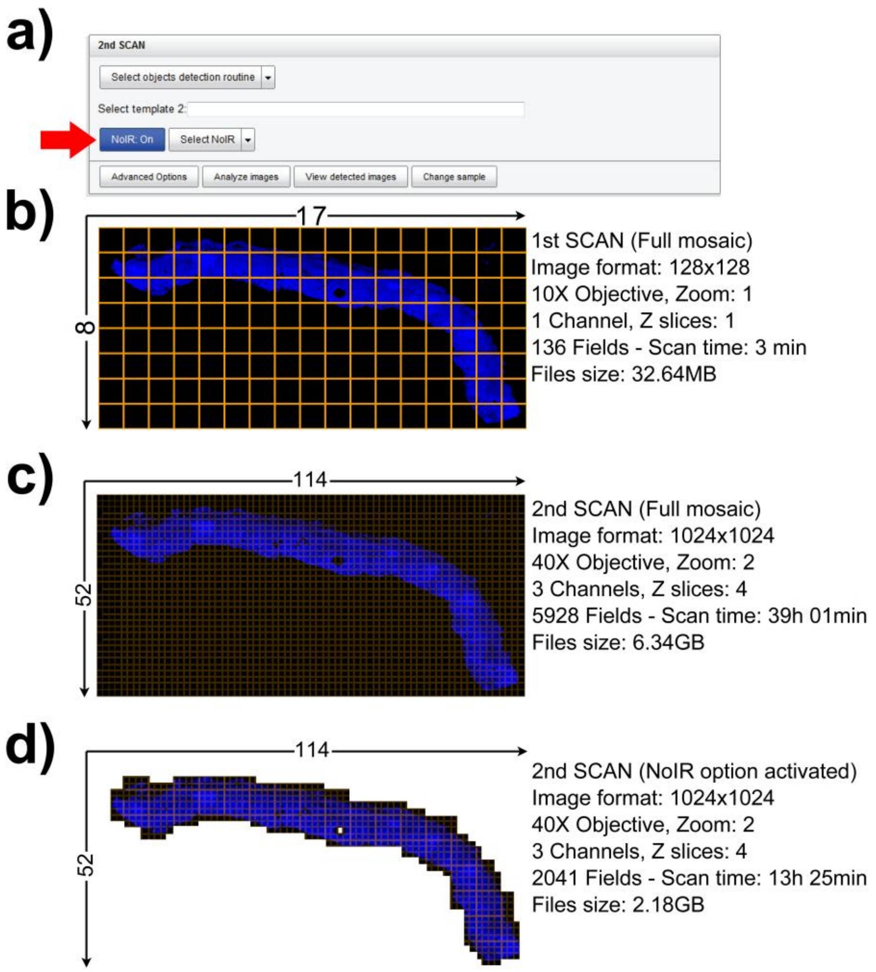

Supplementary Figure S5: *iMSRC*'s Non informative Region (NoIR) tool. When selected (a), each possible field of view of the second scan (orange tiles in c) is analyzed for objects of interest's presence in the searching mosaic (b); fields that are negative for objects of interest are not captured on the second scan (d). c shows the full second scan process (without NoIR) for comparison purposes. NoIR's tool saved up to 26 hours of capturing time and resources by preventing the capture of 3887 tissue empty fields representing 4.2 GB of data (d).

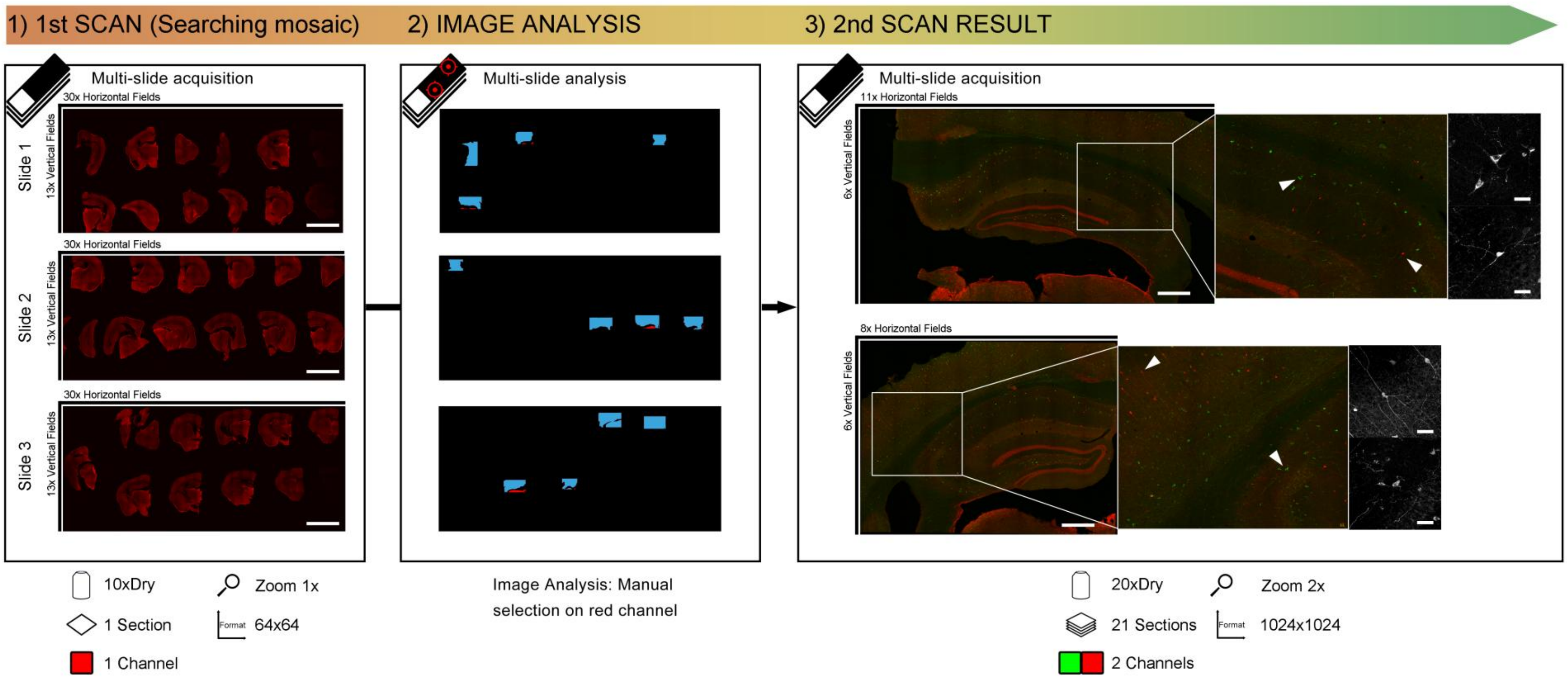

Supplementary Figure S6: *iMSRC* applied to manually-selected, multiple, irregular shape and size, mosaic capturing. Three different slides containing multiple brain slices from mouse were scanned at once at low resolution using an appropriate 3 slides adaptor (1). The resulting searching mosaic images were inspected and hippocampal structures of interest were manually selected (2). Different size and shape mosaics were automatically captured at high resolution for every selected area in step 2; two examples are shown (3). Text and icons show step specifics, see the material and methods section for further details. Scale bars 1: 5 mm, 3: 450 microns, insets: 35 microns.

Supplemental Material and Methods

Automated, multiple, size- and shape-heterogeneous, tissue microarray capturing (Fig. 2b).

Acquisition workflow

The TMA was inspected under the microscope. Searching mosaic settings were chosen to capture a single plane in a DAPI-configured channel using a 10X PL APO 0.4 NA objective and a 256X256 pixel image format. Final capture settings were fixed to capture a ten-section stack in three sequential channels (DAPI, Alexa Fluor 488 and Alexa Fluor 555) using a 40X OIL HCX PL APO 1.25 NA objective, 1.5 zoom and a 1024X1024 pixel image format. Both settings were saved for further work with ***iMSRC***.

- Step 1- searching mosaic capturing: a whole-slide searching mosaic was set up and launched using proprietary capturing software. Capture of the searching mosaic took less than five minutes under previously defined conditions.
- Step 2- image analysis: ***iMSRC***'s pre-existing image analysis routine for tissue detection was used to distinguish tissue from background. The searching mosaic was inspected using imageJ; macro detection parameters were set to detect objects brighter than five arbitrary units (AU) and larger than 1,000 pixels. ***iMSRC*** was then deployed under the previously defined searching mosaic, image analysis routine and final capture settings. The result was a new final capture settings file encoding as many individual mosaics as was needed, each one of which was of the required size to cover a single piece of tissue.
- Step 3- final capture: the final capture settings file was loaded on the proprietary software and capture was launched overnight. As an example, the TMA shown in Figure2 comprised 77 separate pieces of tissue, each one of which consisted of (on average) a 6X6 image mosaic.

Once searching mosaic, final capture settings and image analysis routine were optimized; samples were automatically scanned using ***iMSRC***'s fully automated workflow.

Automated image analysis detection of rare circulating tumour cells (Fig. 2c).

Acquisition workflow

Cells were inspected under the fluorescence microscope. Raw searching mosaic and final capture settings were arrived at based on CK- and CDK45-positive cells. Searching mosaic settings were set to capture a single plane in Hoechst 33342-, FITC- and Cy3-configured sequential channels using a 20X PL APO 0.7 NA objective and a 512X512 pixel image format. Final capture settings were set to capture a ten-section stack in three Hoechst 33342, FITC- and Cy3-configured sequential channels using a 63X OIL HCX PL APO 1.4 NA objective, 2X zoom and a 1024X1024 pixel image format. Both settings were saved for further work with ***iMSRC***.

- Step 1- searching mosaic capturing: a whole-slide searching mosaic was set up and launched using proprietary capturing software. Capturing the searching mosaic took less than five minutes under the conditions previously defined.
- Step 2- image analysis: ***iMSRC***'s pre-existing image analysis routine for cell detection was used to determine the coordinates of the FITC-positive cells only, and macro detection parameters were set to detect objects brighter than 140 arbitrary units (AU) and sized between 13 and 45 pixels. ***iMSRC*** was then deployed under the previous searching mosaic, image analysis routine and final capture settings. The result was a new final capture settings file merging the final capture settings with the objects' coordinates detected using the searching mosaic.
- Step 3- final capture: the final capture settings file was uploaded to the proprietary software. Final capture and searching mosaic settings were refined and the final capture of all positive objects launched.

Once searching mosaic, final capture settings and the image analysis routine were optimized, samples were automatically scanned using ***iMSRC***'s fully automated workflow.

Semi-automated image analysis detection and capturing of several hippocampal structures on different slides (Supplementary Fig. S6).

Cell characterisation of brain nuclei is crucial to unravelling their function and response to illness and treatments.

What is described below is based on unpublished work, and therefore details not of relevance to this paper's purpose have been deliberately omitted.

Adult C57 mice were transcardially perfused with 4% paraformaldehyde. Brains were dissected, post-fixed overnight in the same fixative, rinsed and cryoprotected by immersion in a 30% sucrose solution. Fifty micron cryosections were then prepared using a freezing cryotome, mounted on slides and double immunostained for parvalbumin and somatostatine (1:1000 dilution incubated overnight at room temperature, PV 235. Swant; Switzerland and D-20 sc-7819. Santa Cruz Biotechnology, inc.,USA respectively). The cryosections were then incubated at room temperature for two hours with secondary antibodies coupled to Alexa Fluor 568 and Alexa Fluor 488, Life Technologies).

Samples were analyzed under a microscope driven by ***iMSRC*** tuned to manually select hippocampi, as well as analyze and automatically capture each body (through assigning different size mosaics). Up to three slides with multiple tissue section samples were analyzed simultaneously by way of a three-slide adaptor frame.

The acquisition workflow was as follows:

Samples were inspected under the microscope. Searching mosaic settings were set to capture a single plane in a Alexa 568-configured channel using a 10X PL APO 0.4 NA objective and a 64X64 pixel image format. Final capture settings were fixed to capture a twenty-one-section stack in two Alexa Fluor 594- and Alexa Fluor 488-configured sequential channels using a 20X HCX PL APO 0.7 NA objective, 2 zoom and a 1024X1024 pixel image format. Both settings were saved for further work with ***iMSRC***.

- Step 1- searching mosaic capturing: three whole-slide searching mosaics were set up and launched using proprietary capturing software. Capturing the searching mosaic took less than five minutes using the capture conditions previously described.
- Step 2- image analysis: searching mosaics were inspected under imageJ. Objects of interest were manually selected by drawing a rectangular selection; the rest of the image was set to black. ***iMSRC***'s pre-existing image analysis routine for tissue detection was used to discriminate tissue from background by setting the intensity threshold parameter to 1-255.

iMSRC was then deployed under the previous searching mosaic, image analysis routine and final capture settings. The result was a new final capture settings file encoding as many individual mosaics as needed, each one of which was of the required size to comprise a single hippocampus.

- Step 3- final capture: the final capture settings file was loaded on to the proprietary software. Capture was launched overnight.

Since manual detection did not allow fully automated capturing, subsequent sets of samples were captured by launching every step in a sequential manner, using pre- defined searching mosaic and final capture settings.
